# Tazobactam selects for multidrug resistance

**DOI:** 10.1038/s44259-025-00122-2

**Published:** 2025-05-30

**Authors:** Emma R. Holden, Muhammad Yasir, A. Keith Turner, Ian G. Charles, Mark A. Webber

**Affiliations:** 1 Quadram Institute Bioscience, Norwich Research Park, Norwich, Norfolk NR4 7UQ UK; 2 Centre for Microbial Interactions, Norwich Research Park, Norwich, Norfolk NR4 7UG UK; 3 Norwich Medical School, University of East Anglia, Norwich Research Park, Norwich, Norfolk NR4 7TJ UK

**Keywords:** Target identification, Antimicrobial resistance, Bacterial genes

## Abstract

Piperacillin-Tazobactam is a β-lactam/β-lactamase inhibitor combination that is amongst the most prescribed antimicrobials in hospital medicine. Piperacillin is inactivated by many common beta-lactamases, but tazobactam inhibits these, allowing successful treatment. The effect of piperacillin on Gram-negative bacteria has been widely studied, but less attention has been paid to the effects of tazobactam. We used a massive transposon mutagenesis approach (TraDIS-*Xpress*) to identify genes in *Escherichia coli* that affect survival when exposed to piperacillin and tazobactam, separately and together. We found significant differences between the two drugs: a striking finding was that multiple efflux pump families and regulators were essential for survival in the presence of tazobactam, but only one efflux system was beneficial for piperacillin. Exposure of *Escherichia coli* and *Klebsiella pneumoniae* to piperacillin and/or tazobactam selected for mutants with reduced susceptibility, and tazobactam selected for efflux and cell envelope mutants associated with multidrug resistance. We identified other pathways involved in tazobactam susceptibility, including the shikimate kinase AroK. Genes involved in DNA replication and repair reduced *E. coli* susceptibility to a combination of piperacillin and tazobactam but were not identified after exposure to either drug alone. Tazobactam can select for mutants with increased efflux activity, and the development of future β-lactamase inhibitors should consider potential selective impacts of both inhibitor and antibiotic.

## Introduction

Piperacillin-tazobactam is one of the most prescribed antibiotic treatments in hospital medicine and is on the WHO list of essential medicines. The combination is used to treat a wide range of infections, including pneumonia, urinary tract infections and skin and soft tissue infections^1^. Beta-lactam antibiotics such as piperacillin disrupt peptidoglycan synthesis, an essential component of the bacterial cell envelope. Piperacillin is a ureidopenicillin and resistance to this class of antibiotic can be conferred by genes coding for β-lactamases, which cleave the β-lactam ring, rendering the antibiotic ineffective. To prevent the activity of β-lactamases, β-lactam antibiotics can be given in conjunction with a β-lactamase inhibitor, such as tazobactam. These inhibit β-lactamase enzymes so that the activity of the antibiotic is protected. Extensive research has been conducted on how the cell responds to treatment with β-lactam antibiotics, including piperacillin^2^. However, relatively little work has focussed on how bacteria respond to β-lactamase inhibitors, including tazobactam, and whether these compounds have any significant activity beyond their interactions with β-lactamases^3^.

Large-scale genomic screens have previously been used to identify the extended complement of genes and pathways that affect susceptibility to various antibiotics, and this work has often identified many genes that influence susceptibility beyond the principal target^4^. We have developed one such method, TraDIS-*Xpress*, where massively dense transposon mutant libraries investigate bacterial loci involved in responses to stress. These libraries make use of a transposon-encoded outward-transcribing inducible promoter, which allows changes in gene expression as well as gene disruption to be assayed for roles in survival under a given stress. This provides information about essential genes, which are often targets for antibiotics. We have recently used this approach to identify known and unknown mechanisms of action and resistance to triclosan^5^, fosfomycin^6^, fluoroquinolones^7^, meropenem^8^, trimethoprim and sulfamethoxazole^9^.

In this work, we used TraDIS-*Xpress* to study how *Escherichia coli* responds to piperacillin and tazobactam, separately and in combination. We identified known genes involved in sensitivity to piperacillin, but also found many genes under selective pressure after exposure to tazobactam alone. These included a suite of genes involved in multiple drug resistance with multidrug efflux pumps and regulators, whereas only one efflux system was implicated in piperacillin susceptibility. To explore the implications of these findings, we exposed *E. coli* and *Klebsiella pneumoniae* to both piperacillin and tazobactam and selected resistant mutants to both. Analysis of these resistant mutants revealed tazobactam selected for mutations in genes involved in efflux activity and regulation, and membrane permeability more readily than piperacillin. The demonstration that tazobactam promotes selection of multidrug resistance shows that the impact of β-lactamase inhibitors on target bacteria warrants further study.

## Results

### Multiple efflux systems affect susceptibility to tazobactam, but only AcrAB affects piperacillin susceptibility

The TraDIS-*Xpress* data identified 41 genes that affected the susceptibility of *E. coli* to piperacillin, 74 to tazobactam and 108 to a combination of the two drugs, with 159 genes identified in total from all three conditions (Supplementary Table 1). The variation of sequence reads per insertion site between replicates was low for all conditions tested (Supplementary Fig. 1), indicating a high degree of experimental correlation.

**Fig. 1:**
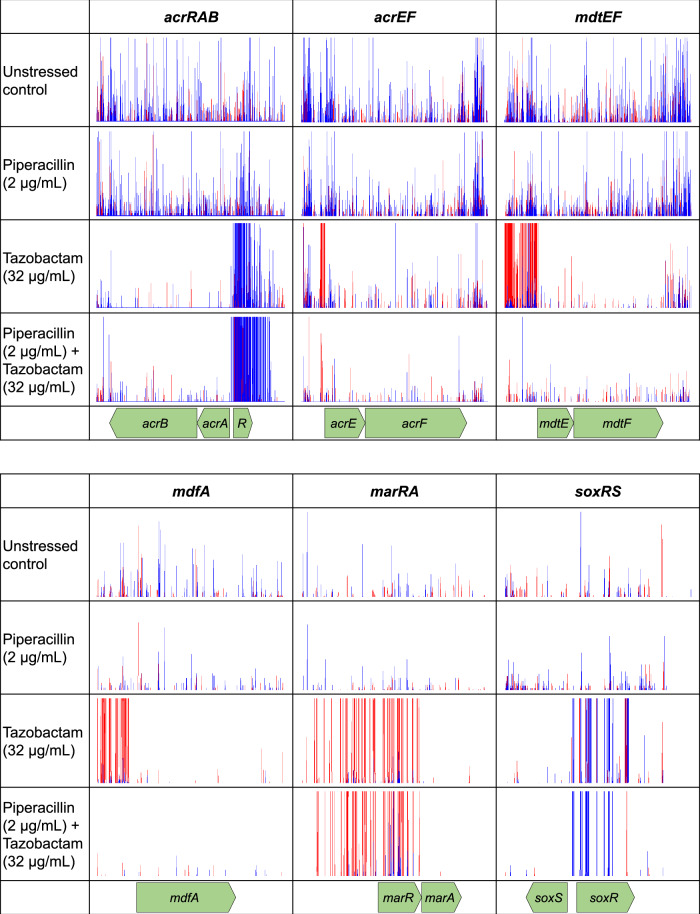
Insertion frequency in and around genes expressing efflux pumps and their regulators in *E. coli* treated with piperacillin and tazobactam, both separately and in combination, relative to an unstressed control. Each vertical line indicates the location of a transposon insertion, red indicates the transposon orientation is left-to-right and blue indicates right-to-left as viewed.

After exposure to tazobactam alone, TraDIS-*Xpress* identified many efflux systems that affected susceptibility. Increased expression of *acrA*, *acrB*, *acrE*, *acrF*, *mdtE*, *mdtF* and *mdfA* appeared to reduce susceptibility to tazobactam, whilst transposon insertions into these genes increased susceptibility (Fig. 1). A similar pattern was seen for systems regulating efflux, increased expression of positive regulators *marA*, *soxS* and *rob* was seen in the presence of tazobactam and transposon insertions within negative regulators of efflux strongly reduced susceptibility to tazobactam. The log_2_-fold differences in insertions in *acrR* (11.8), *marR* (14.9) and *soxR* (15.3) are the highest in this data set, indicating a very strong selective pressure favouring inactivation of these genes in the presence of tazobactam.

In contrast to the tazobactam data, when the library was exposed to piperacillin alone, only *acrR* was found to affect fitness with smaller log_2_-fold changes (up to 2.5) in insertion frequencies after drug exposure (Supplementary Table 1). This data reveals that efflux activity and regulation are extremely important for survival in the presence of tazobactam, but less so for piperacillin. When piperacillin and tazobactam were combined, a similar pattern to the tazobactam alone data was seen with increased expression of *acrA*, *acrB*, *acrE, acrF, marA*, *soxS* and *rob* being beneficial to survival. With the addition of piperacillin, there was no longer the same fitness benefit to overexpressing the *mdtEF* and *mdfA* efflux pumps.

### Other genes impacting efflux function were important in tazobactam susceptibility

Genes with indirect roles in efflux regulation also affected tazobactam susceptibility. These included the Lon protease, which reduces the availability of the efflux activators MarA and SoxS. Following growth in tazobactam there were 7.4 log_2_-fold more insertions in *lon* compared to control conditions, indicating inactivation of Lon improves growth following tazobactam exposure, possibly due to the absence of its protease activity upon MarA and SoxS.

Genes involved in the synthesis of osmoregulated periplasmic glucans, *opgG* and *opgH*, increased tazobactam susceptibility but reduced piperacillin susceptibility. Osmoregulated periplasmic glucans have been previously linked to efflux activity^10^ and are thought to affect the activity of two-component signalling systems^11,12^, which were also found to have a strong effect on tazobactam susceptibility. The PhoPQ signal transduction system reduced tazobactam susceptibility, whilst the CpxAR system increased susceptibility; both signalling systems have also been implicated in efflux activity^10^. Glutathione synthesis has also previously been linked to efflux activity^10,13^, and we found insertions inactivating glutathione synthesis via *gshA* and *gshB* strongly reduced tazobactam susceptibility. Together, we show a consistent signal for the importance of genes encoding efflux systems or efflux regulators when *E. coli* is exposed to tazobactam.

### Tazobactam selects for efflux mutants

**Fig. 2:**
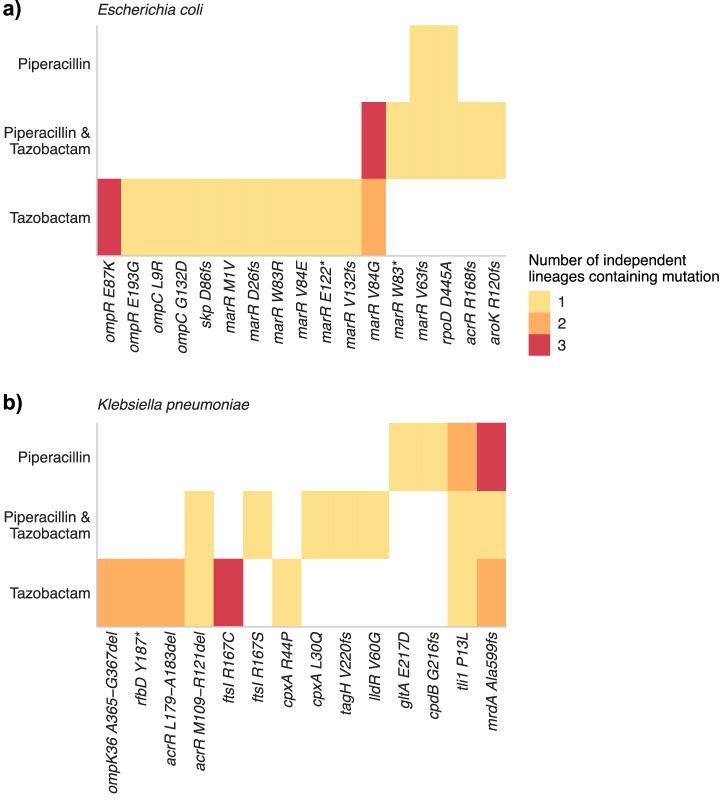
Mutations found in *E. coli* and *K. pneumoniae* following exposure to piperacillin, tazobactam or the two combined. **a** shows data from *E. coli* and **b** shows data from *K. pneumoniae*. Colours show the number of independent lineages where the same mutations was selected. Mutations selected via different methods are combined.

To test the hypothesis that exposure to tazobactam could select for mutations affecting multidrug efflux, we used two approaches. First, we exposed *E. coli* on agar plates supplemented with inhibitory concentrations of piperacillin, tazobactam, or the two combined. We isolated 15 mutants that grew on concentrations of tazobactam above the parental MIC, but could not isolate any mutants from piperacillin, or when both drugs were combined. In parallel, we used an evolution model where we repeatedly exposed *E. coli* in liquid culture to each drug. Each day, the cultures were passaged into fresh LB broth supplemented with double the concentration of drugs from the previous day. The experiments ended after 10 passages where *E. coli* was growing at 128 µg/mL piperacillin (32 times the MIC) and 2048 µg/mL tazobactam (8 times the MIC). Individual colonies were isolated from each mutant population and were genome sequenced alongside the mutants selected from a single exposure to tazobactam to find mutations from the parent strain. Exposure to tazobactam selected nine separate mutations within *marR* across multiple independent lineages, and one mutation in local efflux regulator *acrR*, whereas exposure to piperacillin alone selected a single mutation in *marR* in one lineage (Fig. 2). Tazobactam also selected mutations affecting membrane permeability in *ompC* encoding an outer membrane porin, its regulator *ompR*^14^ and *skp* encoding a periplasmic chaperone involved assembly of OmpC^15^. The combination of piperacillin and tazobactam selected for mutations in *rpoD* encoding sigma factor 70^16^ and *aroK* encoding a shikimate kinase^17^.

To determine if similar selective pressures were exerted by each drug on another pathogenic bacterium, analogous experiments were completed with *Klebsiella pneumoniae*. Mutations in *acrR* were found in multiple independent lineages of *K. pneumoniae* exposed to tazobactam (Fig. 2) along with various mutations in genes with roles in membrane permeability and integrity, including outer membrane porin *ompK36* (analogous to *ompC* in *E. coli*)^14^, *cpxA* involved in regulating membrane integrity in *K. pneumoniae*^18^ and *rfbD* involved in LPS O-antigen biosynthesis^19^. The combination of piperacillin and tazobactam selected mutations in *tagH*, encoding an ATP-binding subunit of an ABC transporter exporting cell wall glycans^20^ and *lldR*, involved in regulating lactate metabolism^21^. Exposure to piperacillin alone selected mutations in *gltA* involved in the TCA cycle and a nucleotide phosphodiesterase encoded by *cpdB*^22^. A mutation in *tli1*, encoding a T6SS immunity protein^23^, was selected for in all three drug conditions.

Together with our findings in *E. coli*, this data confirms that tazobactam can select for changes in efflux and membrane permeability in two important pathogens.

Exposure to tazobactam, both with and without piperacillin selected mutations in *ftsI*, encoding penicillin binding protein 3 (PBP3) in *K. pneumoniae*^24^. This was surprising, as piperacillin has been characterised to bind and inhibit PBP3 encoded by *ftsI*^25,26^, yet we did not see mutations in *ftsI* upon exposure to piperacillin alone, nor did we see a significant change in the fitness of *ftsI* mutants in the TraDIS-*Xpress* data when exposed to piperacillin and tazobactam separately or in combination. We did see a mutation in *mrdA* encoding PBP2 in *K. pneumoniae* exposed to piperacillin and tazobactam separately and in combination, but we did not see a significant change in the TraDIS-*Xpress* data for *mrdA*. The TraDIS-*Xpress* data showed a significant log_2_-fold change in insertions in *mrcB* and *lpoB*, encoding PBP1B and its activator, respectively^24,27^.

### The shikimate kinase AroK had a strong effect on growth and survival in the presence of tazobactam in *E. coli*

**Fig. 3:**
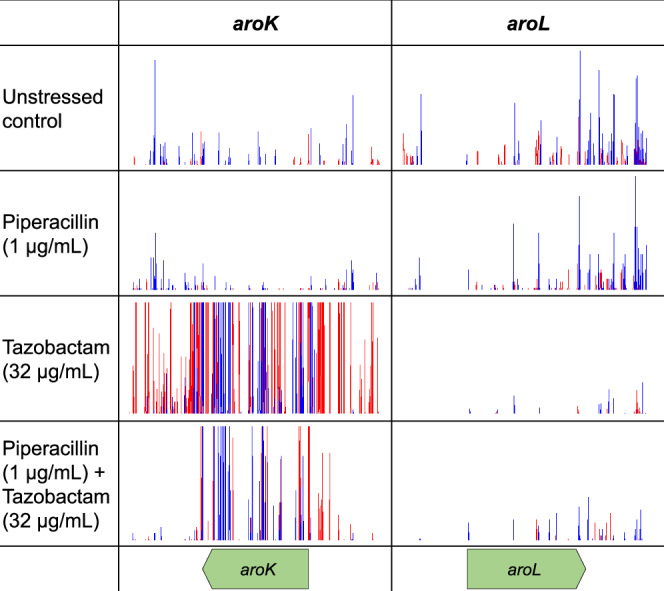
Insertion frequency in and around *aroK* and *aroL* in *E. coli* treated with piperacillin and tazobactam, both separately and in combination, relative to unstressed controls. Red lines indicate the transposon-located promoter is facing left-to-right and blue lines show the promoter facing right-to-left. Images are representative of two independent replicates.

Analysis of the TraDIS-*Xpress* data found a 10.6 log_2_-fold increase in insertion mutations in *aroK* in conditions treated with tazobactam relative to control conditions (Fig. 3), indicating that inactivation of *aroK* was very strongly beneficial for survival in the presence of tazobactam. Supporting this idea, we also found a frameshift variant of *aroK* in an *E. coli* mutant continuously exposed to increasing concentrations of piperacillin and tazobactam (Fig. 2). AroK is a shikimate kinase involved in chorismate biosynthesis, an intermediate in the synthesis of phenylalanine, tyrosine and tryptophan and also a precursor of folic acid, ubiquinone, menaquinone, and enterochelin^28^. AroK is one of two shikimate kinases in *E. coli* alongside AroL, but we saw no signal for insertions impacting *aroL* after tazobactam exposure (Fig. 3). Deletion of *aroK* or *aroL* did not, though, significantly affect tazobactam susceptibility in *E. coli* (data not shown).

### Genes involved in DNA replication, transcription and translation have a common effect on survival against both drugs

There were some genes that affected susceptibility of *E. coli* to both piperacillin and tazobactam: most of these similarities were in genes involved in translation, where their inactivation reduced susceptibility in all conditions relative to unstressed controls. These included genes involved in tRNA modification and fidelity (*truA*^29^, *mnmE* and *mnmG*^30^), ribosome biogenesis, (*bipA*^31^), and *dsbA*, responsible for forming disulphide bonds in periplasmic proteins^32^. We also found *rapA*, important for recycling RNA polymerase^33^, reduced susceptibility to piperacillin and tazobactam, both separately and in combination, when inactivated. These findings suggest that a reduction in transcription rate and translation fidelity may benefit survival to a wide range of stresses and may represent examples of tolerance following a generalised reduction in growth rate^34^.

**Fig. 4:**
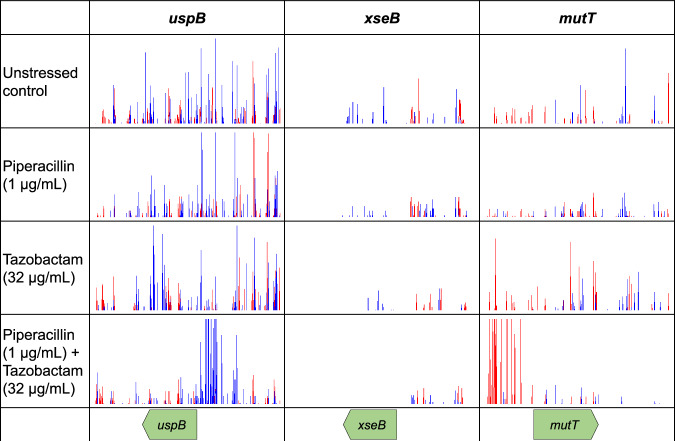
Insertions around genes involved in DNA repair and replication in *E. coli* treated with piperacillin and tazobactam, both separately and in combination, relative to unstressed controls. Red lines indicate the transposon-located promoter is facing left-to-right and blue lines show the promoter facing right-to-left. Images are representative of two independent replicates.

We identified that DNA repair and replication pathways also impacted susceptibility to both piperacillin and tazobactam when combined, but were not identified after exposure to either drug alone. Increased expression of *uspB* involved in DNA recombination repair, and *mutT* involved in maintaining DNA replication fidelity, were both beneficial for survival in the presence of both drugs (Fig. 4). The exonuclease *xseB*, involved in DNA repair, was also essential for survival in the presence of both drugs (Fig. 4) suggesting that the combination of piperacillin and tazobactam elicits greater DNA damage than either drug separately. This highlights a probable synergy between piperacillin and tazobactam independent of β-lactamase inhibition.

## Discussion

By screening the *E. coli* genome, we found that tazobactam elicited a strong efflux response from a wide variety of efflux pump families, their local and global regulators, as well as genes that affect global regulator activity. This was in stark contrast to the much more limited efflux response elicited by piperacillin, which consisted of only the AcrAB efflux pump regulator AcrR. When cells were treated with a combination of piperacillin and tazobactam, the global regulators MarA, SoxS and Rob, and an additional efflux pump AcrEF were beneficial for survival, showing the selective impact of tazobactam is maintained when combined with piperacillin. We also showed that tazobactam readily selects for efflux mutants in *E. coli* and *K. pneumoniae*, two pathogens where efflux-mediated resistance is important clinically. Mutations analogous to those we selected within the efflux regulator *marR* are well characterised and have previously been shown to reduce susceptibility to multiple antibiotics in pathogens^35^. Whilst efflux activity alone may only confer modest reductions in susceptibility to antibiotics, it is clinically important and synergises with other resistance mechanisms^36,37^.

This data demonstrates that a β-lactamase inhibitor which is used to nullify a resistance mechanism may in fact be actively promoting the emergence of another multidrug resistance mechanism. Usage of piperacillin and tazobactam has increased rapidly^38,39^, as have global rates of antimicrobial resistance^40^, so it is extremely important to understand how the antibiotics we use may contribute to resistance.

Tazobactam has been well-characterised as an inhibitor of β-lactamases, but little previous work has studied wider impacts on the bacterial cell. In addition to the evidence for tazobactam to select for increased efflux, we also found a very strong signal for interaction with the shikimate kinase AroK. This may suggest a direct interaction between tazobactam and AroK, as mutants with insertional inactivation of *aroK* showed a considerable benefit in the initial TraDIS-*Xpress* screen, and a mutation within *aroK* was found in an *E. coli* mutant selected after exposure to piperacillin-tazobactam. However, defined mutants in AroK and homologue AroL showed no significant change in susceptibility to tazobactam, suggesting the increase in competitive fitness seen in the initial screen may not translate to a large change in susceptibility. Shikimate is a precursor for the synthesis of aromatic amino acids, enterobactin, folate, ubiquinone and menaquinone: it is possible that reduced synthesis of these compounds slows respiration, carbon metabolism and therefore growth to become tolerant to tazobactam. However, we only see a signal for *aroK* and not *aroL* with the same function, so it is unlikely that this is the mechanism through which reduced tazobactam susceptibility is achieved. AroK has been previously implicated in mecillinam resistance, where deletion reduced susceptibility independently of its shikimate kinase activity, suggesting interactions with β-lactams are possible^28^. It is possible that AroK affects tazobactam susceptibility in the same manner. It is also possible that the combination of reduced AroK activity and increased efflux activity is responsible for a significant reduction in susceptibility to tazobactam. Our current understanding is that multiple genes affect susceptibility to tazobactam, and that these may not form one pathway. Additionally, we understand shikimate kinase activity is complete,x and our validation data supports that the story is not as simple as the TraDIS-*Xpress* data suggests. Further investigation into how tazobactam selects for reduced susceptibility in strains without functional β-lactamases will be interesting to address this question. If tazobactam can select for loss of function of *aroK*, this could also have implications for its susceptibility to mecillinam and other β-lactam antibiotics.

Apart from the major impacts on efflux and *aroK*, we also found many other genes under selective pressure from either tazobactam, piperacillin or the combination of the two. Previous work by Valéria dos Santos et al.^41^ characterised an *E. coli* isolate resistant to piperacillin and tazobactam and found changes to a diverse group of proteins including upregulation of those affecting membrane permeability and DNA stress responses and a lower abundance of proteins involved in respiration and translation. Our work also found genes involved in DNA replication and repair were beneficial after being exposed to both piperacillin and tazobactam but not each drug separately. This and the previous work suggests the combination of both drugs induces DNA damage which may contribute to the synergy between the agents. We also identified multiple genes involved in replication, transcription and translation affected fitness when exposed to piperacillin and tazobactam. Mutations in genes coding for these functions may slow or stop growth, resulting in increased tolerance to the antibiotics in a non-specific manner as has been shown in previous studies^34^

The demonstration here that tazobactam exerts a selective pressure has implications for how future β-lactamase inhibitors are designed. It is extremely important to prevent selection for multidrug resistance which could be an unintended correlate impact from tazobactam use. Multiple novel β-lactam/β-lactamase inhibitor combinations are in development^42,43^ and our work shows consideration of the selective impact of the inhibitor should not be overlooked. It remains to be seen if other β-lactamase inhibitors will have significant selective impacts of their own and this should be an important consideration for development of new combinations which are urgently needed to counter the emergence of resistance.

## Methods

### Bacterial strains and growth conditions

The *E. coli* BW25113 transposon mutant library used in this study contains a pool of over 800,000 different mutants and has been described previously (Yasir et al.^5^). Approximately 10^7^ CFU/mL of this library was added to 1 mL LB Miller broth in a 96-well deep-well plate and supplemented with concentrations of piperacillin around the MIC (MIC was 4 µg/mL piperacillin, concentrations used were ¼x, ½x, 1x and 2x MIC). Tazobactam was either added or omitted from these conditions at a concentration of 32 µg/mL, corresponding to ¼ times the MIC. The Tn*5* transposon used to create this library contains an outward-transcribing *tac* promoter, which was either induced with 0.2 or 1 µM IPTG or left uninduced. Each condition was carried out in duplicate with two antibiotic-free controls. Cultures were grown for 24 h at 37 °C, shaking, and were centrifuged at 3000 x *g* for 10 min to pellet the cells.

### TraDIS-*Xpress* nucleotide sequencing and informatics

Genomic DNA was extracted from cell pellets following the protocol described by Trampari et al.^44^ and quantified using a Qubit HS assay kit (Invitrogen). Genomic DNA was fragmented with the MuSeek DNA fragment preparation kit (ThermoFisher) and purified using AMPure XP beads (Beckman Coulter). DNA fragments were amplified by PCR using customised biotinylated oligonucleotides of nucleotide sequence specific for hybridisation to one transposon end. Biotinylated DNA fragments were then purified using activated beads from the Dynabeads® kilobaseBINDER™ kit (Invitrogen). Following this, the Dynabeads® with bound DNA fragments were used as the template for a second PCR amplification step, using customised oligonucleotides specific for hybridisation to one transposon end and oligonucleotides specific for the MuSeek adapter nucleotide sequences. Dynabeads® were removed from the PCR reactions using a magnetic stand. The resulting PCR products were purified, and fragments of between 300–500 bp in length were applied to a NextSeq 500 using a NextSeq 500/550 High Output Kit v2.5 (75 Cycles) (Illumina).

Output FastQ files from the NextSeq 500/550 were aligned to the *E. coli* BW25113 (CP009273) reference genome using BioTraDIS (version 1.4.3,) incorporating BWA^45^. Data from conditions treated with different concentrations of IPTG were combined to simplify the data interpretation. The tradis_comparison.R command (part of the BioTraDIS toolkit) was used to determine significant differences (*p* < 0.05, after correction for false discovery) in insertion frequencies per gene between control and test conditions. Increased insertion mutations 5’ to genes, which likely signify genes where increased transcription conferred a selective advantage, were identified visually using the Artemis genome browser^46^.

### Selection of drug-resistant mutants and SNP analysis

Mutants were selected via two methods: single exposure of *E. coli* BW25113 to inhibitory concentrations of piperacillin and/or tazobactam, and a stepwise experimental evolution model where *E. coli* and *K. pneumoniae* DSM30104 (ATCC 13883) were continuously exposed to subinhibitory concentrations, which doubled daily with each passage.

Single-exposure mutant selection was achieved by inoculating dense cultures (approximately 10^10^ CFU/mL were prepared by harvesting 10 mL of overnight cultures in LB broth and re-suspending pellets in 100 µL) of *E. coli* on LB agar supplemented with 256 µg/mL Tazobactam. No mutants could be selected with inhibitory concentrations of piperacillin or the combination of piperacillin and tazobactam. Plates were then incubated for 24 h, and mutants recovered. Liquid cultures of mutants were stored in 10% glycerol at −70 °C, and 1 mL of culture was pelleted for DNA extraction.

The stepwise evolution model began with approximately 10^5^ CFU/mL of *E. coli* or *K. pneumoniae* added to 5 mL of LB broth containing 1 µg/mL piperacillin and/or 2 µg/mL tazobactam. These concentrations were subinhibitory for both *E. coli* and *K. pneumoniae*, as the MIC of piperacillin was 4 µg/mL and 16 µg/mL, respectively, and the MIC of tazobactam was 32 µg/mL for both species. Cultures were incubated at 37 °C with shaking at 250 rpm. After 24 h, 100 µL from each condition was transferred into 5 mL fresh LB broth supplemented with piperacillin and tazobactam at double the concentration of the previous condition. At each passage, 1 mL of culture was also collected, pelleted, and stored at −70 °C. Cultures were passaged every 24 h until no growth was detected in each condition and three independent replicates were performed for each condition. The final passage with visible cell growth was diluted and spread on LB agar to grow single colonies. Three single colonies from each condition were chosen to represent each final population and liquid cultures of these mutants were pelleted for DNA extraction.

DNA was extracted and sequenced via Illumina whole genome sequencing following protocols described by Trampari, et al.^44^. FASTQ files generated were compared to the *E. coli* (CP009273) or *K. pneumoniae* (AJJI00000000.1) reference genomes using Snippy version 4.6^47^ to find mutations.

## Supplementary information

220425 PipTazo Supplementary info

## Data Availability

Nucleotide sequence data supporting the analysis in this study have been deposited in ArrayExpress under the accession number E-MTAB-13226. The authors confirm that all supporting data, code and protocols have been provided within the article or through supplementary data files.
