# Supplementary material for: Tazobactam selects for multidrug resistance
Source: NPJ Antimicrob Resist. 2025 May 30;3:48. doi: 10.1038/s44259-025-00122-2 (PMC12125205; doi:10.1038/s44259-025-00122-2)

# Supplementary Data

## **Supplementary table 1**: Genes identified by TraDIS-*Xpress* to have a role in susceptibility of *E. coli* to piperacillin (Pip) and tazobactam (Tazo), either separately or in combination (PipTazo). Log_2_-fold change (Log_2_FC) values shown are statistically significant (*q* < 0.05, *p*-value corrected for false discovery rate) and measure the difference in insertion mutants between antibiotic-treated and untreated (ctrl) conditions. These values could not be calculated for areas outside of genes, and therefore are not shown where insertions denote a change in expression affects fitness. In these cases, the effect of the gene’s expression on fitness is describes in the ‘observed change’ column.

| **Pathway** | **Gene** | **Pip vs ctrl** |  |  | **Tazo vs ctrl** | **PipTazo vs ctrl** |  |  |
| --- | --- | --- | --- | --- | --- | --- | --- | --- |
|  |  | **LogFC**  **(¼ / ½ / 1 / 2xMIC)** | **Observed change** | **LogFC**  **(½ xMIC)** | **Observed change** | **LogFC**  **(¼ / ½ / 1 / 2xMIC)** | **PipTazo vs ctrl**  **Observed change** |  |
| **Efflux pumps & efflux regulators** | *acrA* |  | Increased expression beneficial at sub-MIC concentrations | -1.8 | Fewer insertions & Increased expression beneficial at sub-MIC concentrations |  | Increased expression beneficial at sub-MIC concentrations |  |
|  | *acrB* |  |  | -1.1 | Fewer insertions & Increased expression beneficial at sub-MIC concentrations |  | Increased expression beneficial |  |
|  | *acrR* | 2.5 / 1.2 / - / - | More insertions at sub-MIC concentrations | 11.8 | More insertions | 12.2 / 12.0 / 1.0 / 1.8 | More insertions |  |
|  | *acrE* |  |  |  | Increased expression beneficial |  | Increased expression beneficial at sub-MIC concentrations |  |
|  | *acrF* |  |  |  | Increased expression beneficial |  | Increased expression beneficial at sub-MIC concentrations |  |
|  | *tolC* |  |  | -0.5 | Fewer insertions | -1.3 / - / -1.4 / -2.2 | Fewer insertions |  |
|  | *mprA* |  |  | 3.5 | More insertions |  |  |  |
|  | *mdtE* |  |  |  | Increased expression beneficial |  |  |  |
|  | *mdtF* |  |  | -1.6 | Fewer insertions & Increased expression beneficial |  |  |  |
|  | *mdfA* |  |  |  | Increased expression beneficial |  |  |  |
|  | *marR* |  |  | 14.9 | More insertions | 12.0 / 15.7 / - / - | More insertions |  |
|  | *marA* |  |  | -1.0 | Fewer insertions & Increased expression beneficial |  | Increased expression beneficial at sub-MIC concentrations |  |
|  | *soxR* |  |  | 15.3 | More insertions | 11.6 / 11.8 / - / - | More insertions at sub-MIC concentrations |  |
|  | *soxS* |  |  |  | Increased expression beneficial |  | Increased expression beneficial at sub-MIC concentrations |  |
|  | *rob* |  |  |  | Increased expression beneficial |  | Increased expression beneficial at sub-MIC concentrations |  |
| **Transport across membranes & outer membrane porins** | *chbB* | -1.0 / -1.2 / - / -1.0 | Fewer insertions |  |  |  |  |  |
|  | *trkA* | -1.1 / -/ -/ - | Fewer insertions at sub-MIC concentrations |  |  |  |  |  |
|  | *pitA* |  |  | 5.0 | More insertions | 3.1 / - / 0.9 / 1.7 | More insertions |  |
|  | *ompC* |  |  | -2.3 | Fewer insertions | -1.8 / - / -4.9 / -5.0 | Fewer insertions |  |
|  | *nhaA* |  |  | -2.1 | Fewer insertions |  |  |  |
|  | *sapF* |  |  | -0.5 | Fewer insertions |  |  |  |
|  | *sapD* |  |  | -1.9 | Fewer insertions |  |  |  |
|  | *ptsP* |  |  | 2.5 | More insertions |  |  |  |
|  | *ptsN* |  |  |  | Increased expression beneficial |  |  |  |
|  | *tatA* |  |  |  |  | - / - / 1.1 / 1.5 | More insertions at above MIC concentrations |  |
|  | *tatB* |  |  |  |  | - / - / 1.0 / 1.4 | More insertions at above MIC concentrations |  |
|  | *tatC* |  |  |  |  | - / - / 1.2 / 1.6 | More insertions at above MIC concentrations |  |
|  | *tatD* |  |  |  |  | - / - / -0.6 / -0.6 | More insertions at above MIC concentrations |  |
| **Extracellular polysaccharides (LPS/ ECA)** | *wecA* | - / -0.7 / -0.8 / -0.7 | Fewer insertions |  |  | - / - / -0.8 / -1.7 | Fewer insertions at above MIC concentrations |  |
|  | *wzzE* | - / -0.8 / -1.0 / -0.7 | Fewer insertions |  |  | - / - / -1.5 / -1.6 | Fewer insertions at above MIC concentrations |  |
|  | *wecB* | - / -0.6 / -0.7 / -0.6 | Fewer insertions |  |  | - / - / -1.1 / -1.6 | Fewer insertions at above MIC concentrations |  |
|  | *wecC* | - / -0.9 / -1.1 / -0.6 | Fewer insertions |  |  | - / - / -1.1 / -1.7 | Fewer insertions at above MIC concentrations |  |
|  | *wecD* |  |  |  |  | - / - / -1.8 / -2.3 | Fewer insertions at above MIC concentrations |  |
|  | *wecE* | -0.5 / -0.8 / -0.7 / -0.5 | Fewer insertions |  |  | - / - / -2.1 / -2.8 | Fewer insertions at above MIC concentrations |  |
|  | *wzxE* | -0.9 / -1.1 / -1.0 / -0.9 | Fewer insertions |  |  | - / - / -1.9 / -2.3 | Fewer insertions at above MIC concentrations |  |
|  | *wecF* |  |  |  |  | - / - / -1.7 / -2.3 | Fewer insertions at above MIC concentrations |  |
|  | *wecG* | - / -0.8 / - / - | Fewer insertions |  |  |  |  |  |
|  | *wbbK* | -0.9 / -0.8 / - / -0.9 | Fewer insertions |  |  |  |  |  |
|  | *waaR* |  |  |  |  | - / - / -1.5 / -2.1 | Fewer insertions at above MIC concentrations |  |
|  | *waaS* | -0.7 / -0.4 / -0.1 / -0.3 | Fewer insertions |  |  |  |  |  |
|  | *lpxL* |  |  |  | Increased expression beneficial |  |  |  |
|  | *lptA* |  |  |  |  |  | Increased expression beneficial at above MIC concentrations |  |
|  | *lptB* |  |  |  |  |  | Increased expression beneficial at above MIC concentrations |  |
|  | *lptC* |  |  |  |  |  | Increased expression beneficial at above MIC concentrations |  |
|  | *lptD* |  |  |  |  |  | Increased expression beneficial at sub-MIC concentrations |  |
| **Cell envelope synthesis** | *mrcA* |  |  |  |  | - / - / -1.4 / -1.9 | Fewer insertions at above MIC concentrations |  |
|  | *mrcB* | -2.3 / -2.2 / -2.6 / -2.5 | Fewer insertions |  |  |  | Increased expression beneficial |  |
|  | *lpoA* |  |  |  |  | - / - / -1.3 / -1.6 | Fewer insertions at above MIC concentrations |  |
|  | *lpoB* | -4.0 / -3.6 / -4.7 / -5.2 | Fewer insertions |  |  | - / - / -4.8 / -4.9 | Fewer insertions at above MIC concentrations |  |
|  | *ldcA* | -3.5 / -3.8 / -3.8 / -4.2 | Fewer insertions |  |  | - / - / -4.8 / -4.2 | Fewer insertions at above MIC concentrations |  |
|  | *nlpI* | -2.0 / -1.8 / -1.9 / -2.1 | Fewer insertions |  |  | -2.1 / - / -3.4 / -3.8 | Fewer insertions |  |
|  | *slt* | -3.6 / -3.9 / -3.7 / -4.3 | Fewer insertions |  |  | -1.7 / - / -5.2 / -4.8 | Fewer insertions |  |
|  | *mepS* | - / -1.8 / - / -2.4 | Fewer insertions |  |  |  |  |  |
|  | *mlaA* |  |  | -1.4 | Fewer insertions |  |  |  |
|  | *mlaB* |  |  | -1.2 | Fewer insertions |  |  |  |
|  | *mlaC* |  |  | -1.9 | Fewer insertions |  |  |  |
|  | *mlaD* |  |  | -1.3 | Fewer insertions |  |  |  |
|  | *mlaE* |  |  | -1.1 | Fewer insertions |  |  |  |
|  | *ampG* |  |  |  |  | - / - / -1.8 / -1.7 | Fewer insertions at above MIC concentrations |  |
|  | *rodZ* |  |  |  |  | - / - / -3.0 / -3.9 | Fewer insertions at above MIC concentrations |  |
| **Translation** | *truA* | 1.3 / 1.3 / 1.5 / 1.5 | More insertions | 2.6 | More insertions | 3.8 / - / 1.7 / 2.0 | More insertions |  |
|  | *typA/ bipA* | 0.8 / 0.9 / 1.2 / 1.3 | More insertions | 3.4 | More insertions | 1.3 / - / 1.3 / 2.2 | More insertions at above MIC concentrations |  |
|  | *mnmE* | 0.9 / 0.8 / 1.0 / 1.5 | More insertions | 3.3 | More insertions | 4.3 / - / 2.0 / 2.9 | More insertions |  |
|  | *mnmG* | - / - / 1.0 / 1.4 | More insertions at above MIC concentrations | 2.9 | More insertions | 4.2 / - / 2.0 / 3.0 | More insertions |  |
|  | *tusA* |  |  |  |  |  | Reduced expression beneficial at sub-MIC concentrations |  |
|  | *tusB* |  |  | 4.2 | More insertions | 6.2 / - / - / - | More insertions at sub-MIC concentrations |  |
|  | *tusC* |  |  | 3.3 | More insertions | 5.6 / - / - / - | More insertions at sub-MIC concentrations |  |
|  | *tusD* |  |  | 2.7 | More insertions | 5.1 / - / - / - | More insertions at sub-MIC concentrations |  |
|  | *tufB* |  |  | 2.3 | More insertions | 4.7 / - / - / - | More insertions at sub-MIC concentrations |  |
|  | *glyT* |  |  |  | Increased expression beneficial |  | Increased expression beneficial at sub-MIC concentrations |  |
|  | *fusA* |  |  |  | Increased expression beneficial |  | Reduced expression beneficial at sub-MIC concentrations |  |
|  | *rplK* |  |  |  | Increased expression beneficial |  |  |  |
|  | *thrT* |  |  |  |  | 8.9 / 6.2 / 3.1 / 3.6 | More insertions |  |
|  | *usg* |  |  |  |  | 2.8 / - / 1.1 / 1.2 | More insertions |  |
|  | *deaD* |  |  |  |  | 2.3 / - / 0.7 / 0.7 | More insertions |  |
|  | *prfC* |  |  |  |  | 1.2 / - / 2.6 / 2.6 | More insertions |  |
|  | *prmB* |  |  |  |  | - / - / 2.5 / 2.9 | More insertions at above MIC concentrations |  |
| **Two component signalling systems** | *cpxA* |  |  | 1.9 | More insertions | 5.6 / - / 0.4 / 0.9 | More insertions |  |
|  | *cpxR* |  |  | 3.1 | More insertions |  | Increased expression beneficial at sub-MIC concentrations |  |
|  | *phoP* |  |  | -0.6 | Fewer insertions | - / - / -1.1 / - | Fewer insertions |  |
|  | *phoQ* |  |  | -2.4 | Fewer insertions | -1.1 / - / -1.5 / -2.4 | Fewer insertions |  |
|  | *rcsB* | - / -0.9 / -1.6 / -1.6 | Fewer insertions |  |  |  | Increased expression beneficial at sub-MIC concentrations |  |
|  | *arcB* |  |  | -0.8 | Fewer insertions |  |  |  |
|  | *envZ* |  |  |  |  | -1.1 / - / -2.2 / -2.4 | Fewer insertions |  |
|  | *ompR* |  |  |  |  | - / - / -3.4 / -2.4 | Fewer insertions at above MIC concentrations |  |
|  | *mzrA* |  |  | 3.9 | More insertions |  |  |  |
|  | *nlpE* |  |  |  |  |  | Increased expression beneficial |  |
|  | *safA* |  |  |  |  |  | Increased expression beneficial at sub-MIC concentrations |  |
| **DNA housekeeping, replication & repair** | *rssA* | - / -1.1 / -0.9 / -1.0 | Fewer insertions |  |  |  |  |  |
|  | *dcd* | 2.3 / 2.4 / - / - | More insertions at sub-MIC concentrations |  |  |  |  |  |
|  | *maoP* |  |  | 3.2 | More insertions |  |  |  |
|  | *fis* |  |  |  | Increased expression beneficial |  |  |  |
|  | *ihfB* |  |  |  |  | - / - / -6.9 / -5.5 | Fewer insertions at above MIC concentrations |  |
|  | *xseB* |  |  |  |  |  | Fewer insertions |  |
|  | *mutT* |  |  |  |  |  | Increased expression beneficial |  |
|  | *uspB* |  |  |  |  |  | Increased expression beneficial |  |
| **Cell division** | *tolQ* |  |  |  |  | - / - / -5.1 / -3.2 | Fewer insertions at above MIC concentrations |  |
|  | *tolR* | -2.5 / -2.7 / -3.0 / - | Fewer insertions |  |  | - / - / -3.3 / -5.5 | Fewer insertions at above MIC concentrations |  |
|  | *tolA* | -3.5 / -3.3 / -3.2 / -3.5 | Fewer insertions |  |  | - / - / -5.0 / -4.7 | Fewer insertions at above MIC concentrations |  |
|  | *tolB* | -3.3 / -3.3 / -2.9 / -3.5 | Fewer insertions |  |  | - / - / -9.6 / -9.3 | Fewer insertions at above MIC concentrations |  |
|  | *pal* | -1.9 / - / - / - | Fewer insertions at sub-MIC concentrations |  |  | - / - / - / -7.5 | Fewer insertions at above MIC concentrations |  |
|  | *damX* |  |  | 1.9 | More insertions |  |  |  |
|  | *ftsE* |  |  |  |  | -1.8 / -3.0 / -2.1 / -2.1 | Fewer insertions |  |
|  | *ftsN* |  |  |  |  | - / - / -1.7 / -2.0 | Fewer insertions at above MIC concentrations |  |
|  | *ftsX* |  |  |  |  | - / - / -2.0 / -2.3 | Fewer insertions at above MIC concentrations |  |
|  | *minC* |  |  |  |  | -1.1 / - / -2.8 / -2.4 | Fewer insertions |  |
|  | *minD* |  |  |  |  | -1.2 / - / -2.3 / -2.0 | Fewer insertions |  |
|  | *minE* |  |  |  |  | - / - / -2.8 / -3.3 | Fewer insertions |  |
|  | *envC* |  |  |  |  | - / - / -1.2 / -0.9 | Fewer insertions at above MIC concentrations |  |
|  | *amiB* |  |  |  |  | 1.7 / - / - / - | More insertions at sub-MIC concentrations |  |
|  | *hflD* |  |  |  |  |  | Increased expression beneficial at sub-MIC concentrations |  |
| **Proteases** | *lon* | - / -0.9 / - / - | Fewer insertions at sub-MIC concentrations | 7.4 | More insertions | 4.7 / - / - / - | More insertions at sub-MIC concentrations |  |
|  | *prc* | -2.0 / -2.3 / -2.4 / -2.5 | Fewer insertions |  |  | - / - / -4.0 / -4.2 | Fewer insertions at above MIC concentrations |  |
|  | *smpB* | 2.1 / 2.1 / 2.6 / 2.3 | More insertions |  |  | - / - / 2.8 / 3.7 | More insertions at above MIC concentrations |  |
|  | *rhlB* |  |  | 2.7 | More insertions |  |  |  |
|  | *degP* |  |  |  |  |  | Increased expression beneficial |  |
| **Chaperones** | *dsbA* | - / - / 1.3 / 1.6 | More insertions at above MIC concentrations | 3.0 | More insertions | 8.6 / 5.3 / 2.3 / 2.8 | More insertions |  |
|  | *dsbB* |  |  |  |  | 3.0 / - / - / - | More insertions at sub-MIC concentrations |  |
|  | *surA* |  |  | -0.2 | Fewer insertions & Reduced expression beneficial |  |  |  |
|  | *ibpB* |  |  | -1.7 | Fewer insertions |  |  |  |
|  | *ibpA* |  |  | -0.6 | Fewer insertions |  |  |  |
|  | *tig* |  |  | -2.0 | Fewer insertions |  |  |  |
|  | *bepA* |  |  |  | Increased expression beneficial |  |  |  |
| **Transcription** | *rapA* | 1.3 / 1.2 / 1.1 / 0.9 | More insertions | 4.1 | More insertions | 8.6 / 4.2 / 1.1 / - | More insertions at sub-MIC concentrations |  |
|  | *greA* |  |  | 6.0 | More insertions |  |  |  |
|  | *nusA* |  |  |  | Increased expression beneficial |  |  |  |
| **Respiration, Electron transport & ATP synthesis** | *atpB* | - / - / -2.1 / - | Fewer insertions at sub-MIC concentrations &  Increased expression beneficial at above MIC concentrations |  |  |  | Increased expression beneficial |  |
|  | *pgi* |  |  | -1.2 | Fewer insertions |  |  |  |
|  | *cra* |  |  |  |  | 2.8 / - / 1.4 / 1.8 | More insertions |  |
|  | *cydB* |  |  |  |  | - / - / 2.2 / 2.1 | More insertions at above MIC concentrations |  |
| **Amino acid metabolism** | *aroK* |  |  | 10.6 | More insertions | 6.1 / 3.3 / - / 2.5 | More insertions |  |
|  | *dapF* | -1.7 / - / - / - | Fewer insertions at sub-MIC concentrations |  |  |  |  |  |
|  | *glnD* |  |  |  |  | 2.9 / - / 0.9 / 1.1 | More insertions |  |
| **Stress response transcription factors & regulation** | *rpoS* |  |  | -2.0 | Fewer insertions | -2.2 / - / -1.8 / -3.3 | Fewer insertions & Increased expression beneficial |  |
|  | *yheO* |  |  |  |  | 3.1 / - / - / - | More insertions at sub-MIC concentrations |  |
|  | *lrp* |  |  | 2.5 | More insertions |  |  |  |
| **Fe-S cluster synthesis & repair** | *rseC* |  |  | 3.2 | More insertions | 1.1 / - / - / - | More insertions at sub-MIC concentrations |  |
|  | *iscS* |  |  |  |  |  | Increased expression beneficial at sub-MIC concentrations |  |
|  | *iscR* |  |  |  |  | 4.4 / - / -1.2 / - | More insertions at sub-MIC concentrations |  |
|  | *hscA* |  |  |  |  | - / - / 2.0 / 2.4 | More insertions at above MIC concentrations |  |
|  | *hscB* |  |  |  |  | - / - / 2.9 / 3.0 | More insertions at above MIC concentrations |  |
| **Glutathione biosynthesis** | *gshA* |  |  | 8.4 | More insertions |  |  |  |
|  | *gshB* |  |  | 4.8 | More insertions |  |  |  |
| **Osmoregulated periplasmic glucans** | *opgG* |  |  | 5.4 | More insertions | - / - / 1.4 / 1.9 | More insertions at above MIC concentrations |  |
|  | *opgH* | -0.7 / - / - / - | Fewer insertions at sub-MIC concentrations | 4.9 | More insertions | - / - / 0.8 / 1.3 | More insertions at above MIC concentrations |  |
| **Toxin-Antitoxin systems** | *higA* | -2.5 / -2.5 / - / - | Fewer insertions at sub-MIC concentrations |  |  |  |  |  |
|  | *dinQ* | - / - / - / 2.2 | More insertions at above MIC concentrations |  |  |  |  |  |
| **Acid stress** | *yodD* |  |  | -3.6 | Fewer insertions |  |  |  |
|  | *ydeP* |  |  |  |  |  | Increased expression beneficial at sub-MIC concentrations |  |
| **Small regulatory RNAs** | *hfq* |  |  |  | Increased expression beneficial |  |  |  |
| **Motility** | *hdfR* |  |  | 2.9 | More insertions |  |  |  |
| **cAMP** | *cyaA* |  |  |  |  | 2.8 / - / - / - | More insertions at sub-MIC concentrations |  |
| **Fimbriae-like protein** | *ydeQ* |  |  |  |  |  | Increased expression beneficial at sub-MIC concentrations |  |
| **Unknown** | *ychJ* | - / -1.2 / -1.3 / -1.3 | Fewer insertions |  |  |  |  |  |
|  | *ybcK* |  |  |  | Increased expression beneficial |  |  |  |
|  | *ybcM* |  |  |  | Increased expression beneficial |  |  |  |
|  | *yidQ* |  |  | 3.5 | More insertions |  |  |  |
|  | *ycbC* |  |  |  |  | - / - / -2.2 / -2.6 | Fewer insertions at above MIC concentrations |  |
|  | *yobH* |  |  |  |  |  | Increased expression beneficial at sub-MIC concentrations |  |

## **Supplementary figure 1:** Insertion frequencies per gene for *E. coli* treated with **a)** piperacillin, **b)** tazobactam, and **c)** a combination of both drugs, relative to unstressed controls. Black points represent the insertion frequency per gene for each replicate to show variation between the replicates, and coloured points show the insertion frequency per gene between the control (x-axes) and each condition (y-axes).


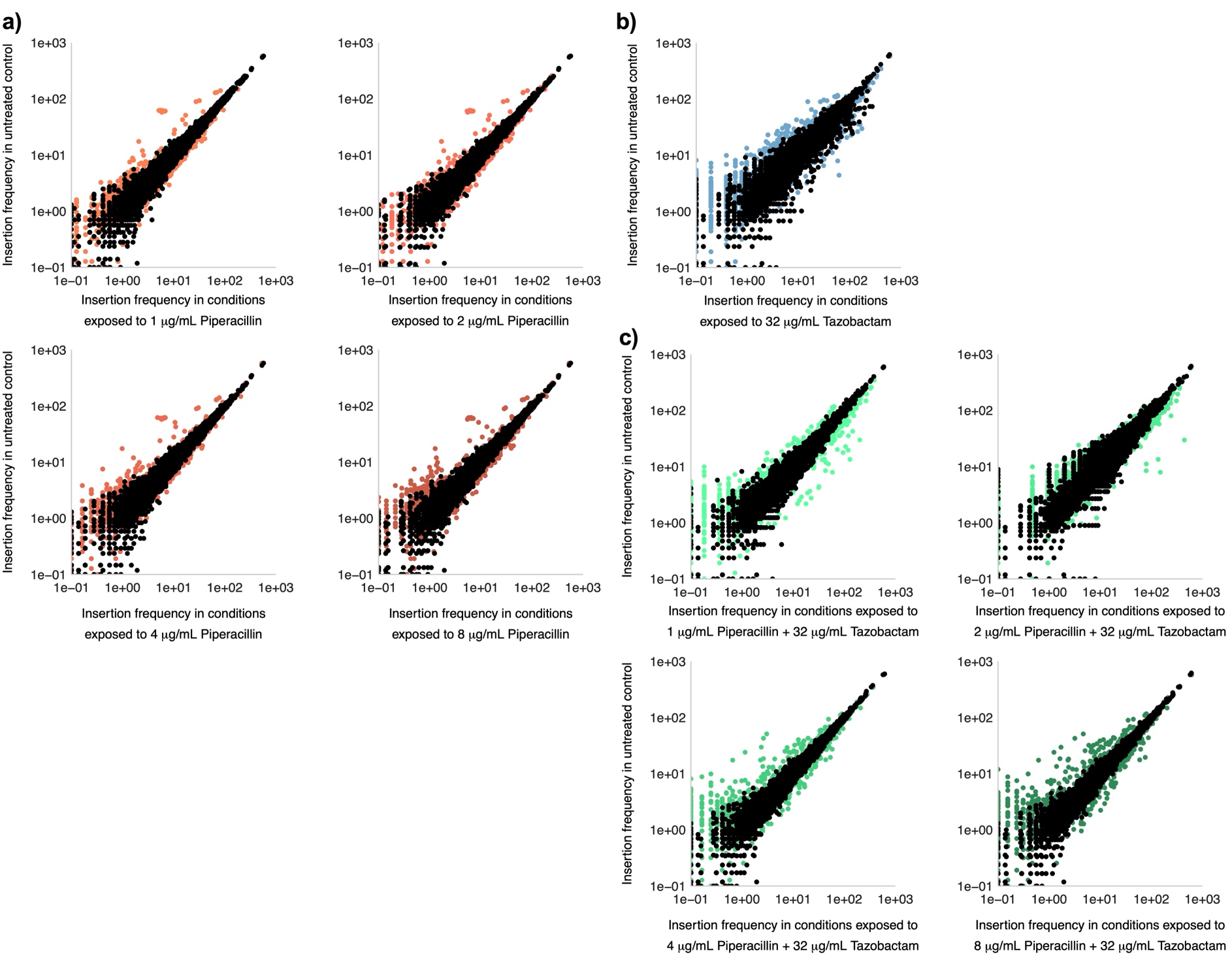

Supplement: Supplementary file 1 — 220425 PipTazo Supplementary info [file 44259_2025_122_MOESM1_ESM.docx]
